# Poor nutritional status is associated with incomplete functional recovery in elderly patients with mild traumatic brain injury

**DOI:** 10.3389/fneur.2023.1131085

**Published:** 2023-04-04

**Authors:** Bingcheng Zhu, Yunwei Ou, Xufei Guo, Weiming Liu, Liang Wu

**Affiliations:** ^1^Department of Neurosurgery, Beijing Tiantan Hospital, Capital Medical University, Beijing, China; ^2^China National Clinical Research Center for Neurological Diseases, Beijing, China; ^3^Neurological Center, People's Hospital of Ningxia Hui Autonomous Region, Yinchuan, China

**Keywords:** mild traumatic brain injury, geriatric nutritional risk index, nutrition, elderly, prognosis, nutritional supplement

## Abstract

**Background:**

The geriatric nutritional risk index (GNRI) is a simple index for evaluating the nutrition status of elderly patients. Many investigations have demonstrated that this index is associated with the prognosis of several diseases. This study aims to identify the relationship between the GNRI and recovery in elderly mild traumatic brain injury (mTBI) patients.

**Methods:**

A total of 228 mTBI patients older than 65 years were included in this study. mTBI was defined as an injury to the brain with a loss of consciousness of 30 min or less, a duration of posttraumatic amnesia of <24 h, and an admission Glasgow Coma Scale (GCS) score of 13–15. The Glasgow Outcome Scale Extended (GOSE), an outcome scale assessing functional independence, work, social activities, and personal relationships, was applied to assess the recovery of the patients. The clinical outcome was divided into complete recovery (GOSE = 8) and incomplete recovery (GOSE ≤ 7) at 6 months after the injury. Multivariate logistic regression was applied to evaluate the association between the GNRI and recovery of elderly mTBI patients, with adjustment for age, sex, hypertension, diabetes, and other important factors.

**Results:**

The receiver operating curve (ROC) analysis demonstrated that the cutoff value of GNRI was 97.85, and the area under the curve (AUC) was 0.860. Compared to the patients with a high GNRI, the patients with a low GNRI were older, had a higher prevalence of anemia, acute subdural hematoma, and subarachnoid hemorrhage, had a higher age-adjusted Charlson Comorbidity Index value, and had lower levels of albumin, lymphocytes, and hemoglobin. Multivariable analysis showed that high GNRI was associated with a lower risk of 6-month incomplete recovery (OR, 0.770, 95% CI: 0.709–0.837, *p* < 0.001).

**Conclusion:**

The GNRI has utility as part of the objective risk assessment of incomplete 6-month functional recovery in elderly patients with mTBI.

## Introduction

Traumatic brain injury (TBI), with an incidence of 349 per 10,000 individuals each year, is a major cause of death and disability among adults (1, 2). The clinical severity of TBI is stratified based on the Glasgow Coma Scale (GCS) score into mild TBI (GCS score of 13–15), moderate TBI (GCS score of 9–12), and severe TBI (GCS score of 3–8) (3). Mild TBI (mTBI) is the most common type of TBI, accounting for nearly 90% of all TBI cases (4, 5). Compared with other age groups, adults older than 65 years are at higher risk of suffering from mTBI (6).

Several residual impairments, such as physical symptoms, cognitive deficits, and behavioral disturbances, emerge after mTBI (4). Although most patients will recover completely within weeks to months, there remains a subgroup of patients who suffer persistent symptoms that affect work and life (7). According to van der Naalt et al., one in three patients recovers incompletely at 6 months after mTBI (8). Moreover, Jacobs et al. reported that the risk of poor recovery will increase among older mTBI patients (9). Rothweiler et al. also found that over 60% of younger patients had a favorable outcome 1 year after mTBI compared to < 20% in older patients over 60 years (10).

With growing aging populations, the incidence of mTBI among elderly patients will increase significantly (11). Finding prognostic predictive factors and improving the clinical outcome of geriatric mTBI patients can effectively relieve the cost of healthcare, but few investigations have focused on this point. Malnutrition is common among geriatric populations and has been considered to be associated with a worse prognosis for many diseases among elderly patients (12, 13). Therefore, we hypothesize that malnutrition also has an adverse effect on the recovery of elderly mTBI patients. The geriatric nutritional risk index (GNRI), calculated by actual weight, ideal weight, and serum albumin concentration, is a simple and accurate index that reflects the nutritional condition of elderly patients (14, 15). Furthermore, Su et al. identified that GNRI was also correlated with the clinical outcome of geriatric patients suffering from moderate to severe TBI (16). However, the correlation between the GNRI and the clinical outcome of elderly mTBI patients is still unclear. In this study, we aimed to explore the association between the GNRI and the clinical outcome of elderly mTBI patients.

## Methods

### Patients

The retrospective study included elderly mTBI patients who presented to Beijing Tiantan Hospital from April 2013 to August 2018. The inclusion criteria included the following: 1. Patients who were older than 65 years; 2. Patients who were diagnosed with mTBI, defined by an admission GCS score of 13–15, with posttraumatic amnesia lasting < 24 h and loss of consciousness for < 30 min (8); 3. Patients who did not receive craniotomy before the admission; 4. Patients who did not have a prior diagnosis of an operative neurosurgical condition; and 5. Patients whose peripheral blood test was performed within 24 h after injury. The exclusion criteria were as follows: 1. Patients who regularly took anti-inflammatory glucocorticoids or other drugs affecting the peripheral blood 6 months before the injury; 2. Patients who failed to obtain accurate values of weight and height; and 3. Patients with spinal cord injuries, spine injuries, rib injuries, or injuries on tissues or organs in anatomical regions other than the brain.

### Data collection

The baseline characteristics included age, sex, GCS score on admission, hypertension, diabetes, heart disease, history of cancer, anemia, application of anticoagulant or antiplatelet medicine, current smoking, and drinking. According to the medical record, participants with a history of stroke did not have documented sequelae, suggesting a major influence on normal life before the injury, so we record the history of stroke as well. The age-adjusted Charlson Comorbidity Index (aCCI), a tool evaluating the impact of age and comorbidities on disease progression, was calculated by the formula provided by Koppie et al. (17). The comorbidity-polypharmacy score (CPS), calculated as the absolute sum of the number of preadmission medications plus all known comorbidities, was also collected (18). The data of surgical debridement were included in this study as well. The laboratory test included blood urea nitrogen (Bun), glucose (Glu), albumin (Alb), white blood cell (WBC), lymphocyte (Lym), neutrophil, and hemoglobin (Hgb). All blood markers were measured from the same peripheral blood sample, which was taken from patients within 24 h after injury. The number of patients who underwent neurosurgical interventions during hospitalization and the injury mechanism was also collected. Abnormalities shown on brain computed tomography (CT) scans performed on the day of admission, such as acute subdural hematoma (ASDH), acute epidural hematoma (AEDH), traumatic subarachnoid hemorrhage (TSAH), and intracranial hemorrhage, and skull fracture, were also included in our research. Body mass index (BMI) was calculated as follows: BMI (kg/m^2^) = height/weight^2^. The GNRI was calculated as follows: GNRI = 1.489^*^albumin (g/L) + 41.7^*^ [present body weight (kg)/ideal body weight (kg)]. The ideal body weight for a man was calculated as follows: height (cm) – 100 – [(height (cm)-150)/4]. The ideal body weight for a woman was calculated as follows: height (cm) – 100 – [(height (cm)-150)/2.5].

### Outcome measurement

The patients included in this investigation were followed up at 6 months after injury. The Glasgow Outcome Scale Extended (GOSE), ranging from 1 to 8, was identified to be an effective tool to measure functional outcomes (19). A structured, validated questionnaire was applied to evaluate the GOSE (20). At 6 months after injury, we contacted patients by telephone to complete the questionnaire. If patients failed to complete the questionnaire, we contacted their caregivers or families to evaluate the functional outcome. A lower GOSE score indicated a worse recovery. In this study, GOSE was applied to evaluate the outcome of the patients. The clinical outcome was divided into incomplete recovery (GOSE score of 1–7) and complete recovery (GOSE score of 8).

### Statistical analysis

The distribution of measurement variables was justified by the Kolmogorov–Smirnov test. The normally distributed variables are shown as the mean ± standard deviation (SD) and non-normally distributed data are presented as the median (25–75th percentile). The differences in baseline characteristics between the complete recovery group and the incomplete recovery group were compared by Student's *t*-test, the Mann-Whitney *U*-test, and the chi-square test. A receiver operating characteristics (ROC) curve was utilized to obtain the optimal cuto? value of the GNRI. Then all patients were divided into a low GNRI group and a high GNRI group based on the cutoff value. Multivariate logistic regression models were used to assess the association between GNRI level and incomplete recovery. Net reclassification improvement (NRI) and integrated discrimination improvement (IDI) are two newly emerging indices evaluating the improvement in model performance accomplished by adding new factors to the conventional model (21). A conventional model containing age, sex, smoking, drinking, BMI, hypertension, diabetes, heart disease, history of stroke, history of anticoagulant use, aCCI, anemia, ASDH, and TSAH was established. The risk factors in the conventional model were selected based on previous research (8, 18, 22–26). IDI and NRI were calculated to identify whether adding the GNRI to the conventional model could improve the predictive ability for incomplete recovery. Finally, we also used a restricted cubic spline (RCS) with three knots placed at the 5, 50, and 95th percentiles of the distribution of GNRI. A *P*-value of < 0.05 was considered to be statistically significant. All statistical analyses in this study were performed using SPSS software 22.0 (SPSS Inc., Chicago, IL) and R software [version 4.2.2 (https://www.r-project.org/)].

## Results

### Patient enrollment

**Figure 1:**
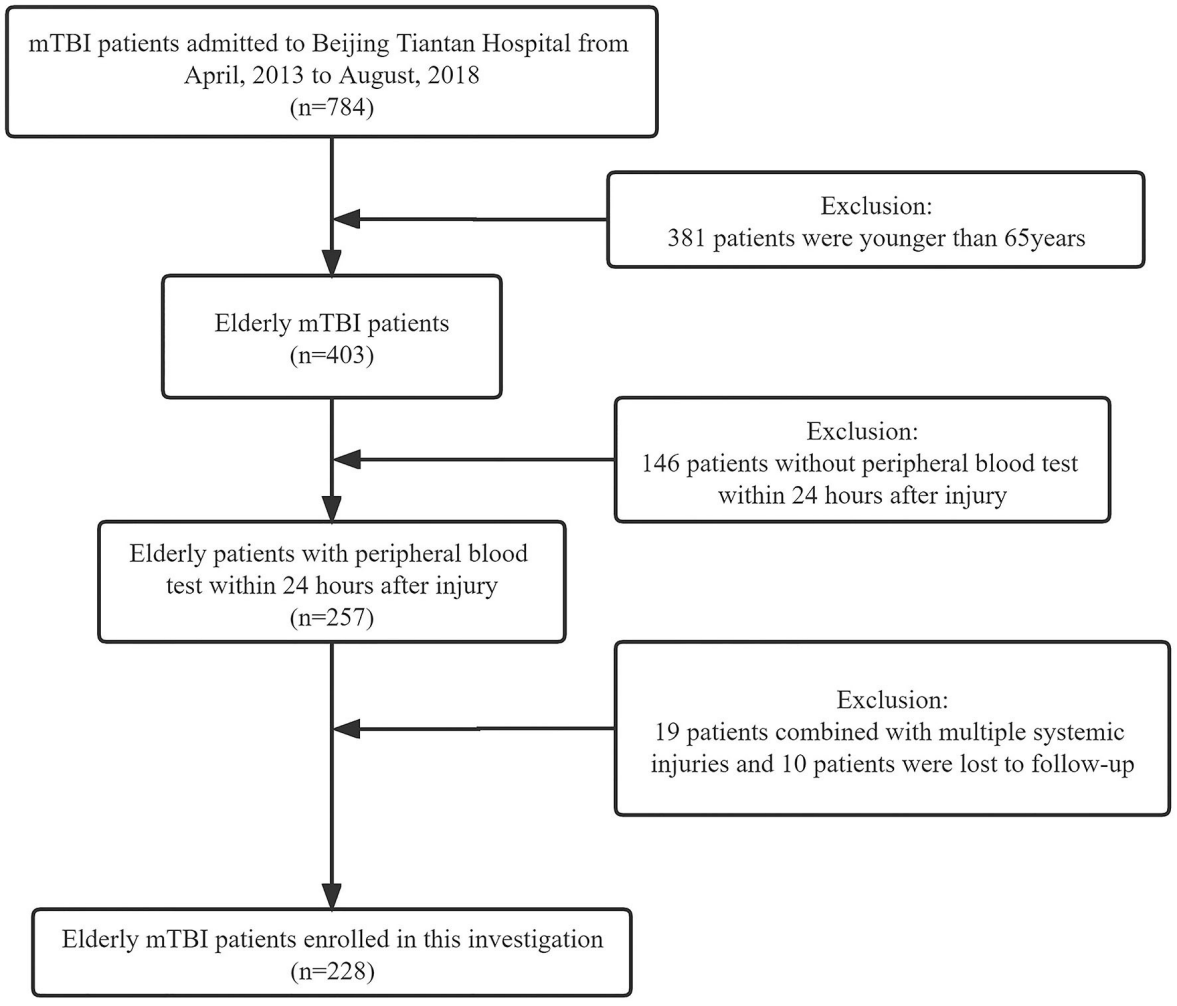
Flow diagram of the study participants. mTBI, mild traumatic brain injury.

From April 2013 to August 2018, a total of 784 mTBI patients were admitted to Beijing Tiantan Hospital. A total of 381 patients who were younger than 65 years were excluded. Among the 403 patients who were older than 65 years, 146 patients did not receive peripheral blood tests within 24 h after the injury. A total of 19 patients had multiple systemic injuries, and 10 patients were lost to follow-up. Therefore, a total of 228 patients were enrolled in this study (Figure 1). None of the patients received neurosurgical treatment for the intracranial lesion or skull fracture, and 10 patients received surgical debridement.

### The receiver operating curve of GNRI

**Figure 2:**
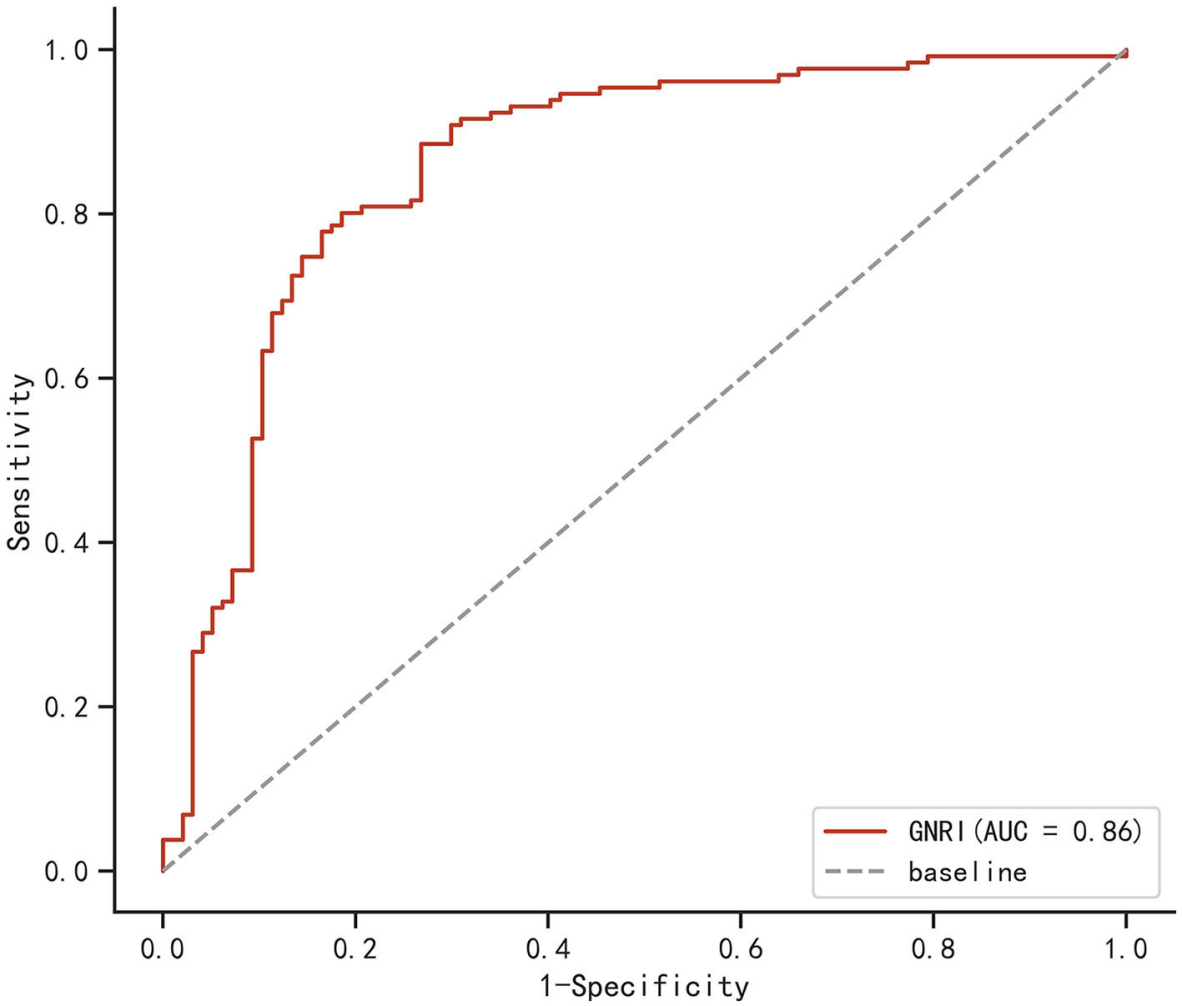
Receiver operating characteristic curve (ROC) of the geriatric nutritional risk index (GNRI) for the prediction of poor recovery in elderly mild traumatic brain injury (mTBI) patients. The area under the curve (AUC) for the GNRI was 0.860 (95% CI: 0.806–0.911, *p* < 0.001; sensitivity: 88.5%, specificity: 73.2%; cutoff value: 97.85).

The ROC curve of the GNRI to predict the clinical outcome of elderly mTBI patients is shown in Figure 2. The ROC curve showed that the area under the curve (AUC) was 0.860 (95% CI: 0.806–0.911, *p* < 0.001). Based on the maximal Youden index (sensitivity + specificity – 1), we found that the optimal GNRI cutoff value as a predictor was 97.85, with a sensitivity of 88.5% and specificity of 73.2%. The results indicated that the GNRI had satisfactory performance in predicting the clinical outcome of elderly mTBI patients.

### Comparison of baseline characteristics

The enrolled patients included 138 men and 90 women. Among these patients, 131 patients recovered completely, and 97 patients recovered incompletely at 6 months after the injury. Table 1 reveals the differences in baseline characteristics between the incomplete recovery group and the complete recovery group. Compared with the incomplete recovery group, patients in the complete recovery group tended to be younger (73.42 ± 6.83 vs. 76.76 ± 7.44, *p* = 0.001), had a lower prevalence of anemia (10/131 vs. 35/97, *p* < 0.001), had lower scores of aCCI (4.53 ± 1.24 vs. 5 (4–6), *p* = 0.008), CPS (3.03 ± 2.22 vs. 3 (1–5), *p* < 0.001), and a lower BMI (22.83 ± 1.30 vs. 23.53 ± 1.84, *p* = 0.001). For the laboratory test, the complete recovery group had higher levels of Alb (39.57 ± 3.67 vs. 36.06 ± 2.62, *p* < 0.001) and Hgb (131.34 ± 15.67 vs. 122.00 (104.00–134.00), *p* < 0.001). The GNRI level of the complete recovery group was also significantly higher than that of the incomplete recovery group (101.89 ± 5.09 vs. 94.09 ± 6.08, *p* < 0.001). ASDH was more likely to appear on the CT of the incomplete recovery group (67/131 vs. 68/97, *p* = 0.004).

**Table 1:** Comparison of baseline characteristics between complete recovery and incomplete recovery mild traumatic brain injury patients.

**Characteristics**	**Complete recovery (*n* = 131)**	**Incomplete recovery (*n* = 97)**	***P***
**Demographic data**			
Age (year)	73.42 ± 6.83	76.76 ± 7.44	**0.001**
Sex (male: female)	79:52	59:38	0.937
**Personal history**			
Hypertension, *n* (%)	91 (69.46)	63 (64.94)	0.471
Diabetes, *n* (%)	35 (26.72)	21 (21.65)	0.379
Heart disease, *n* (%)	19 (14.50)	18 (18.56)	0.412
History of stroke, *n* (%)	21 (16.03)	18 (18.56)	0.616
Cancer, *n* (%)	8 (6.11)	9 (9.28)	0.367
Anemia, *n* (%)	10 (7.63)	35 (36.08)	**< 0.001**
History of anticoagulant, *n* (%)	1 (0.76)	8 (8.24)	**0.004**
History of antiplatelet, *n* (%)	26 (19.85)	17 (17.52)	0.658
Smoking, *n* (%)	37 (28.24)	26 (26.80)	0.810
Drinking, *n* (%)	25 (19.08)	15 (15.46)	0.477
aCCI	4.53 ± 1.24	5 (4–6)	**0.008**
CPS	3.03 ± 2.22	3 (1–5)	**< 0.001**
BMI, kg/m^2^	22.83 ± 1.30	23.53 ± 1.84	**0.001**
**Laboratory test**			
Bun, mmol/L	5.85 ± 2.32	5.60 (4.50–7.40)	0.105
Glu, mmol/L	8.07 ± 3.52	7.58 ± 3.92	0.325
Alb, g/L	39.57 ± 3.67	36.06 ± 2.62	**< 0.001**
WBC count, 10^9^/L	10.91 ± 3.73	10.00 ± 4.16	0.086
Lym count, 10^9^/L	1.22 ± 0.54	1.06 ± 0.57	0.025
Neutrophil count, 10^9^/L	9.22 ± 3.94	8.32 ± 4.11	0.095
Hgb, g/L	131.34 ± 15.67	122.00 (104.00–134.00)	**< 0.001**
GNRI	101.89 ± 5.09	94.09 ± 6.18	**< 0.001**
**Injury mechanism**, ***n*** **(%)**			
Traffic accident	31 (23.66)	15 (15.46)	0.127
Fall	94 (71.75)	76 (78.35)	0.258
Collison	8 (8.24)	6 (6.18)	0.975
**Brain CT findings**, ***n*** **(%)**			
ASDH	67 (51.14)	68 (70.10)	**0.004**
AEDH	20 (15.27)	9 (9.28)	0.179
TSAH	58 (44.27)	54 (55.67)	0.089
Intracranial hemorrhage	71 (54.20)	55 (56.70)	0.707
Skull fracture	47 (35.88)	35 (36.08)	0.991
Surgical debridement, *n* (%)	3 (2.29)	7 (7.22)	0.072
Glasgow Coma Scale	14.56 ± 0.51	14.59 ± 0.69	0.054

aCCI, age-adjusted Charlson Comorbidity Index; CPS, comorbidity-polypharmacy score; BMI, body mass index; Bun, blood urea nitrogen; Glu, glucose; Alb, albumin; WBC, white blood cell; Lym, lymphocyte; Hgb, hemoglobin; GNRI, geriatric nutritional risk index; ASDH, acute subdural hematoma; AEDH, acute epidural hematoma; TSAH, traumatic subarachnoid hemorrhage. The bold values indicate the values of *p* < 0.05.

**Figure 3:**
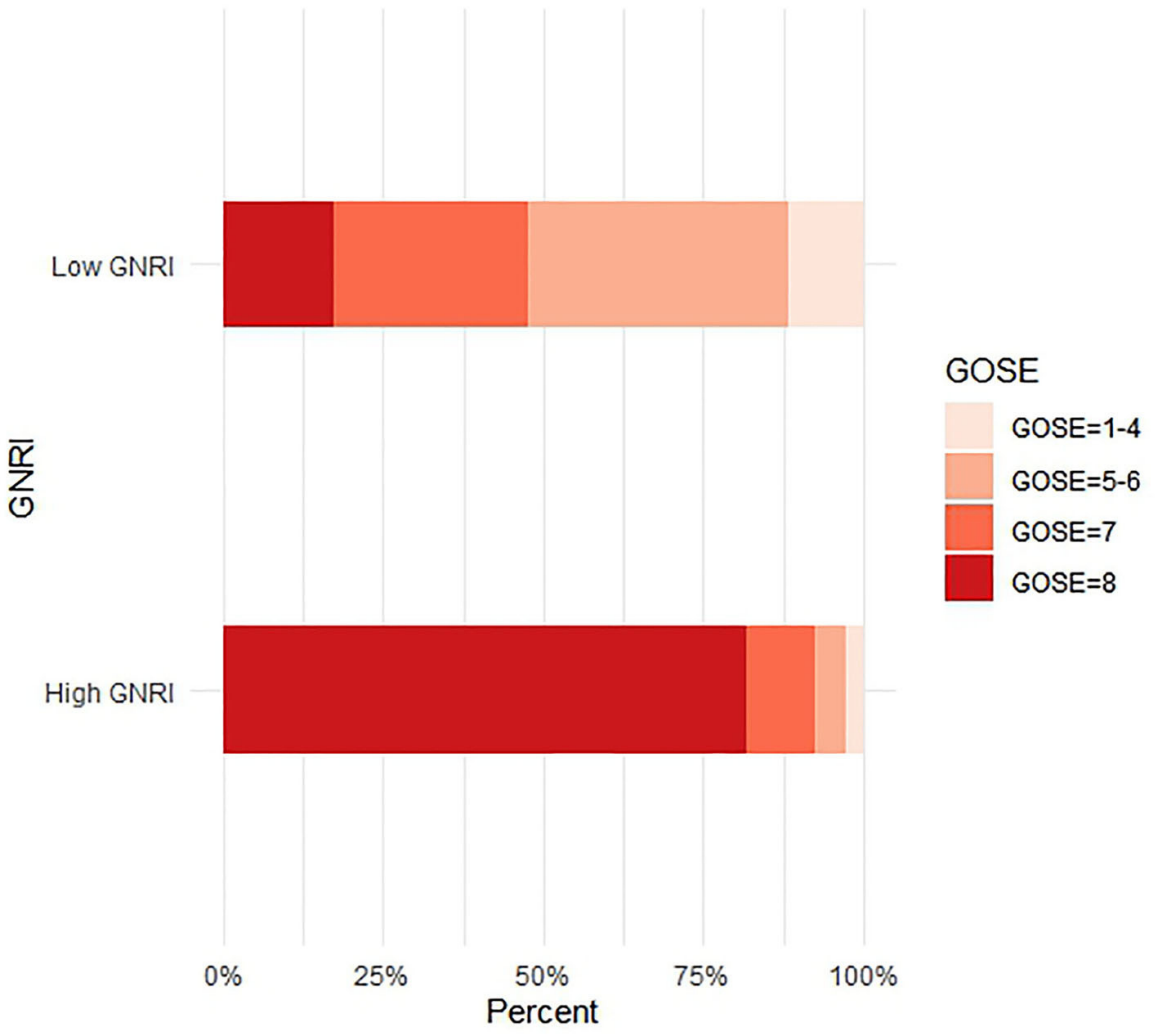
The primary outcome measure was assessed with the use of the GOSE, an outcome scale assessing functional independence, work, social and leisure activities, and personal relationships. The eight outcome categories are death (GOSE = 1), vegetative state (GOSE = 2), lower severe disability (GOSE = 3), upper severe disability (GOSE = 4), lower moderate disability (GOSE = 5), upper moderate disability (GOSE = 6), lower good recovery (GOSE = 7), and upper good recovery (GOSE = 8).

We also divided the patients into a low GNRI group (GNRI < 97.85) and a high GNRI group (GNRI ≥ 97.85) based on the cutoff value. Figure 3 is the stacked bar chart of GOSE 6 months after the injury. Overall, the patients with a low GNRI had a worse recovery. Table 2 shows the comparison of baseline characteristics between the low GNRI and the high GNRI groups. Compared with patients in the low GNRI group, the high GNRI group tended to be younger (73.30 ± 6.55 vs. 77.38 ± 7.72, *p* = 0.001); had a higher prevalence of anemia (10/142 vs. 35/86, *p* < 0.001), collision (9/142 vs. 15/86, *p* = 0.008), ASDH (75/141 vs. 57/86, *p* = 0.046), and TSAH (58/141 vs. 51/86, *p* = 0.011); and had higher levels of Alb (39.87 ± 2.53 vs. 35.22 ± 3.43, *p* < 0.001), Lym (1.25 ± 0.59 vs. 0.99 ± 0.44, *p* < 0.001), and Hgb (130.10 ± 16.71 vs. 120.02 ± 20.54, *p* < 0.001).

**Table 2:** Comparison of baseline characteristics between high GNRI patients and low GNRI patients.

**Characteristics**	**High GNRI (*n* = 142)**	**Low GNRI (*n* = 86)**	***P***
**Demographic data**			
Age (year)	73.30 ± 6.55	77.38 ± 7.72	**0.001**
Sex (male: female)	85:57	53:33	0.791
**Personal history**			
Hypertension, *n* (%)	94 (71.75)	60 (69.77)	0.577
Diabetes, *n* (%)	40 (28.17)	16 (18.60)	0.104
Heart disease, *n* (%)	20 (14.08)	17 (19.77)	0.259
History of stroke, *n* (%)	23 (16.20)	16 (18.60)	0.640
Cancer, *n* (%)	10 (7.04)	7 (8.14)	0.365
Anemia, *n* (%)	15 (10.56)	30 (34.88)	**< 0.001**
History of anticoagulant, *n* (%)	9 (6.34)	6 (6.98)	0.849
History of antiplatelet, *n* (%)	28 (19.72)	15 (17.44)	0.670
Smoking, *n* (%)	40 (28.17)	23 (26.74)	0.816
Drinking, *n* (%)	23 (16.20)	17 (19.77)	0.492
aCCI	4.56 ± 1.35	5 (4.00–6.00)	**0.016**
CPS	2.00 (1.00–4.00)	3.00 (1.00–5.00)	0.337
BMI, kg/m^2^	23.05 ± 1.44	23.26 ± 1.81	0.340
**Laboratory test**			
Bun, mmol/L	5.96 ± 2.65	5.70 (4.47–7.42)	0.232
Glu, mmol/L	7.98 ± 3.29	7.64 ± 4.29	0.499
Alb, g/L	39.87 ± 2.53	35.22 ± 3.43	**< 0.001**
WBC count, 10^9^/L	10.72 ± 3.72	10.20 ± 4.27	0.331
Lym count, 10^9^/L	1.25 ± 0.59	0.99 ± 0.44	**< 0.001**
Neutrophil count, 10^9^/L	8.91 ± 3.74	8.71 ± 4.47	0.720
Hgb, g/L	130.10 ± 16.71	120.02 ± 20.54	**< 0.001**
**Injury mechanism**, ***n*** **(%)**			
Traffic accident	33 (23.24)	13 (15.12)	0.138
Fall	100 (70.42)	68 (79.07)	0.151
Collison	9 (6.34)	15 (17.44)	**0.008**
**Brain CT findings**, ***n*** **(%)**			
ASDH	75 (52.82)	57 (66.28)	**0.046**
AEDH	22 (15.49)	7 (8.14)	0.106
TSAH	58 (40.84)	50 (58.14)	**0.011**
Intracranial hemorrhage	79 (55.63)	47 (54.65)	0.885
Skull fracture	50 (35.21)	32 (37.21)	0.760
Glasgow Coma Scale at admission	14.74 ± 0.54	14.59 ± 0.69	0.099
Surgical debridement, *n* (%)	6 (4.22)	4 (4.65)	0.879
Complete recovery	116 (81.69)	15 (17.44)	**< 0.001**

aCCI, age-adjusted Charlson Comorbidity Index; CPS, Comorbidity-polypharmacy score; BMI, body mass index; Bun, blood urea nitrogen; Glu, glucose; Alb, albumin; WBC, white blood cell; Lym, lymphocyte; Hgb, hemoglobin; GNRI, geriatric nutritional risk index; ASDH, acute subdural hematoma; AEDH, acute epidural hematoma; TSAH, traumatic subarachnoid hemorrhage. The bold values indicate the values of *p* < 0.05.

### GNRI levels and recovery of mTBI

**Figure 4:**
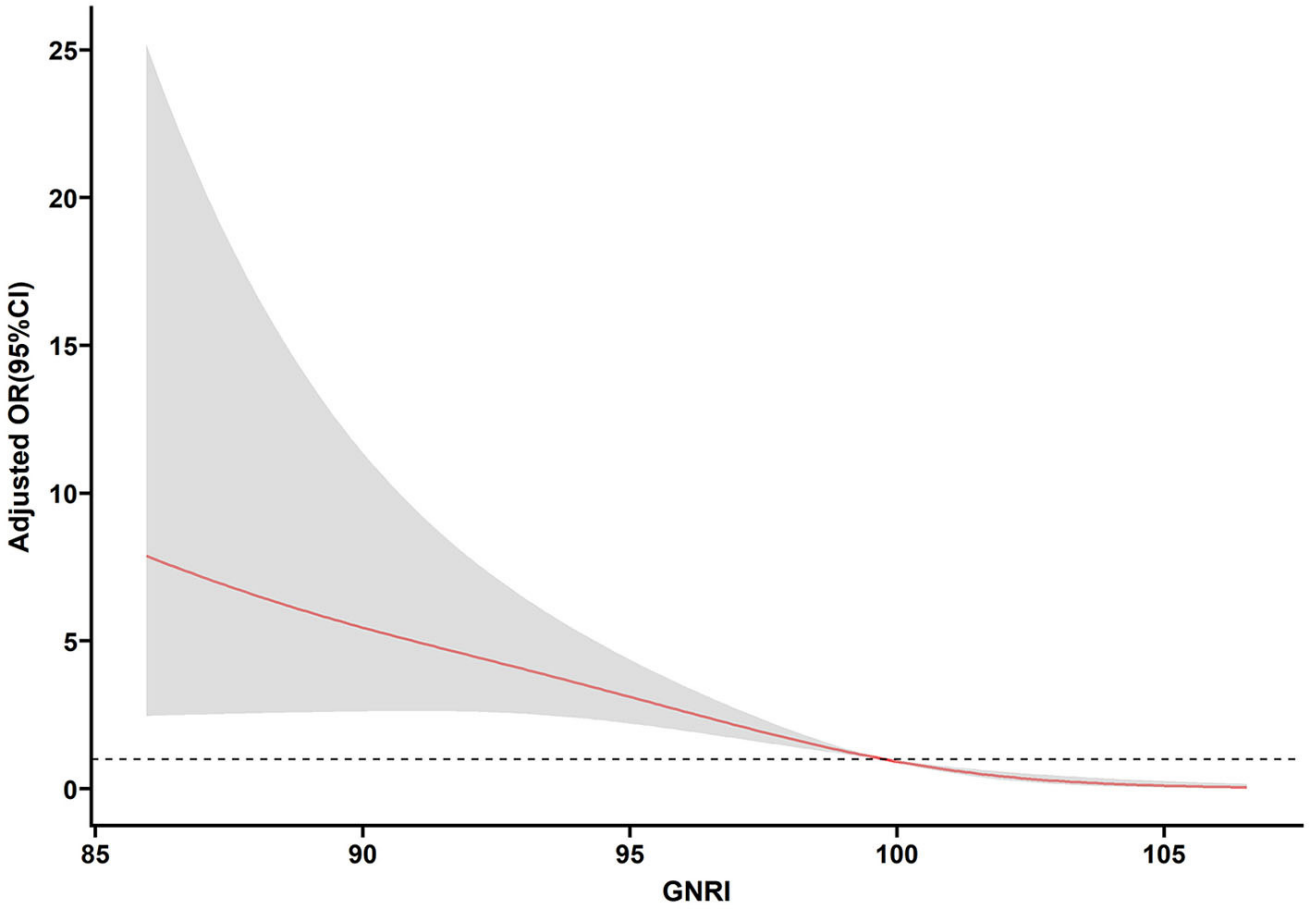
Association of the GNRI and poor recovery in elderly mTBI patients. Odds ratios and 95% confidence intervals derived from restricted cubic spline regression (*p* for non-linearity = 0.108), with three knots placed at the 5, 50, and 95th percentiles of the distribution of GNRI. Odds ratios were adjusted for the variables included in the fully adjusted model in Table 3.

Table 3 shows the association between the GNRI and the risk of incomplete recovery for elderly mTBI patients. After adjusting for age, sex, drinking, smoking, hypertension, diabetes, heart disease, history of stroke, cancer, history of anticoagulant use, history of antiplatelet use, anemia, ASDH, aCCI, CPS, Lym, and Hgb, the risk of incomplete recovery decreased with each increment in GNRI levels (OR, 0.770, 95% CI: 0.709–0.837, *p* < 0.001). When the GNRI was evaluated as two tertiles based on the cutoff value, compared with the low GNRI (GNRI < 97.85), the fully adjusted odds ratio (OR) for the risk of incomplete recovery in the high GNRI level (GNRI ≥ 97.85) was 0.047 (0.020–0.109, *p* < 0.001). Moreover, we also made a multivariable-adjusted RCS (Figure 4). Figure 4 shows that the GNRI had a linear association with incomplete recovery (*p* < 0.001, *p* for non-linearity = 0.108).

**Table 3:** Association between the baseline GNRI and the risk of incomplete recovery.

	**The number of events (incomplete recovery), *n* (%)**	**Crude model**		**Minimally adjusted model**		**Fully adjusted model**	
		**OR (95% CI)**	***P*** **-value**	**OR (95% CI)**	***P*** **-value**	**OR (95% CI)**	***P*** **-value**
**All patients**							
GNRI	97	0.769 (0.715–0.827)	**< 0.001**	0.780 (0.722–0.844)	**< 0.001**	0.770 (0.709–0.837)	**< 0.001**
**GNRI level**							
Low GNRI	71	1.0 (Ref)		1.0 (Ref)		1.0 (Ref)	
High GNRI	26	0.052 (0.025–0.105)	**< 0.001**	0.820 (0.737–0.912)	**< 0.001**	0.047 (0.020–0.109)	**< 0.001**

The crude model included age and sex. The minimally adjusted model was adjusted for age, sex, drinking, smoking, hypertension, diabetes, heart disease, history of stroke, cancer, history of anticoagulant use, history of antiplatelet use, anemia, and acute subdural hematoma. The fully adjusted model was adjusted for all the variables in the minimally adjusted model plus the age-adjusted Charlson Comorbidity Index, comorbidity-polypharmacy score, lymphocyte, and hemoglobin level.

OR, odds ratio; CI, confidence interval; GNRI, geriatric nutritional index. The bold values indicate the values of *p* < 0.05.

### Incremental prognostic value of the GNRI

The NRI and IDI were calculated to evaluate whether adding the GNRI to a conventional model could improve the risk prediction of incomplete recovery for elderly mTBI patients. As shown in Table 4, adding the GNRI to conventional risk factors improved the risk reclassification for incomplete recovery (NRI: 126.17%, *p* < 0.001; IDI: 25.00%, *p* < 0.001).

**Table 4:** Reclassification and discrimination statistics for mild traumatic brain injury by geriatric nutritional risk index at baseline.

	**Continuous NRI, %**		**IDI, %**	
	**Estimate (95% CI)**	***P*** **-value**	**Estimate (95% CI)**	***P*** **-value**
Conventional model	Ref		Ref	
Conventional model + GNRI (continuous)	126.17 (105.66–146.67)	**< 0.001** ^*^	25.00 (19.50–30.51)	**< 0.001** ^*^

The conventional model included age, sex, smoking, drinking, body mass index, hypertension, diabetes, heart disease, history of stroke, history of anticoagulant, age-adjusted Charlson Comorbidity Index, anemia, acute subdural hematoma, and traumatic subarachnoid hemorrhage.

^*^Adding the GNRI to the conventional model improved the risk reclassification for incomplete recovery significantly (NRI: 126.17%, p < 0.001; IDI: 25.00%, p < 0.001).

NRI, net reclassification improvement; IDI, integrated discrimination improvement; CI, confidence interval; Ref, reference. The bold values indicate the values of *p* < 0.05.

## Discussion

In this research, we found that elderly patients with a high GNRI were more likely to achieve complete recovery. The ROC analysis demonstrated that the GNRI had a satisfactory performance for predicting incomplete recovery. Moreover, adding the GNRI to a conventional model could significantly improve the risk prediction of incomplete recovery.

Malnutrition is a common status among elderly patients and is mainly caused by different comorbidities and decreased food intake (27–29). At present, the importance of nutrition assessment is increasing, and many previous investigations have suggested that the evaluation of nutritional status should be performed as a part of clinical management for hospitalized elderly patients (30, 31). In 2005, Bouillanee et al. demonstrated that the GNRI was an effective tool to evaluate the nutritional risk for elderly medical patients for the first time (14). Since then, the GNRI has been researched in many studies and is considered to be more representative and stable than other nutritional assessment tools. Some investigations found that the GNRI had a stronger correlation with many nutritional indices, such as the circumference of the mid-upper arm muscle and the arm muscle area, which meant that the GNRI had a better reflection of systemic nutritional status (32–34). In addition, previous studies also demonstrated that the GNRI was less likely to be affected than some nutritional serum biomarkers, such as Alb (35). Moreover, compared with the Mini Nutritional Assessment (MNA) or other nutritional questionnaires, the GNRI was easier to be obtained from elderly patients who were difficult to communicate with. Therefore, the GNRI was more suitable to be applied in clinical practice to screen the nutritional conditions in elderly populations. Recently, an increasing number of investigations reported that GNRI has a high predictive ability for the prognosis of elderly hospitalized patients. Ruan et al. reported that the GNRI was an independent prognostic factor for elderly patients with cancer cachexia (36). Seoudy et al. found that a decreased GNRI increased all-cause mortality of elderly patients undergoing transcatheter aortic valve replacement (37). Kregel et al. also identified that a low GNRI was associated with high mortality and increased infectious complaints among geriatric trauma patients (34). Moreover, the GNRI was identified to have satisfactory performance in predicting the clinical outcome of elderly severe TBI patients as well (16).

In our investigation, the cutoff value of the GNRI was 97.85, and the patients were divided into a low GNRI group and a high GNRI group based on this value. According to Bouillanee et al., patients with a GNRI of < 98 were considered to have malnutrition (14). This present study identified that the patients in the low GNRI group (< 97.85) were more likely to suffer a worse clinical outcome, which meant that malnourished elderly mTBI patients might have a higher prevalence of poor recovery. There are several potential pathological mechanisms to explain our findings. Aquilani et al. reported that malnutrition inhibited protein synthesis and glucose utilization in the brain tissue, which might adversely affect the rehabilitation of intracranial hemorrhagic foci caused by mTBI and contribute to a worse recovery (38). Meanwhile, previous investigations also identified that malnutrition was correlated with an increased risk of white matter hyperintensities, microbleeds, and brain atrophy among geriatric populations, which could lead to poor recovery as well (39, 40). Moreover, serum Alb, an important part of the GNRI, was also reported to have an anti-oxidative stress effect, which could improve the neurological recovery after mTBI (41–43). Therefore, compared with elderly patients with no risk of malnutrition, malnutritional geriatric patients may suffer an increased risk of poor recovery after mTBI.

Some previous research studies explored the relationship between nutritional status and clinical outcomes after TBI. Li et al. reported that nutritional status was associated with the clinical outcome for severe TBI patients (44). Wang et al. also found that malnutritional status increased mortality and the risk of worse clinical outcomes for TBI patients (45). Okazaki et al. identified that elderly severe TBI patients with low Alb levels were more likely to have an unfavorable outcome (46). Most studies have focused on moderate to severe TBI patients, but few studies investigated elderly mTBI patients. Our investigation reveals that nutrition plays an important role in the recovery of geriatric mTBI populations. Considering that malnutritional elderly patients were more likely to suffer a worse clinical outcome, we hypothesize that regular nutritional assessment and nutritional intervention will show a beneficial effect on recovery for elderly patients after mTBI. Previous studies demonstrated that nutritional supplementation is a critical means of improving recovery for severe TBI patients (47, 48). Intravenous zinc and branched-chain amino acid infusions were identified to decrease the risk of mortality and disability after severe TBI (47, 49). Lee et al. and Razmkon et al. also reported that intramuscular vitamin D and vitamin E injections contributed to a better resolution of cognitive symptoms and a better post-TBI clinical outcome. However, single-nutrient supplementation was also considered to have some side effects, making it difficult to generalize to older mTBI patients (5). Compared with single-nutrient supplementation, daily diets are complex and contain numerous substances that often act synergistically (50). Moreover, some dietary patterns, such as the Mediterranean diet, have been identified as effective nutritional interventions to improve the prognosis of many diseases (51–54). Therefore, we consider that daily diet can play an important role in improving the recovery of mTBI, and more investigations are needed to explore suitable dietary patterns for malnutritional geriatric mTBI patients.

## Limitations

Our investigation recognizes the following limitations. First, this is a retrospective analysis, which may bring some inevitable bias. The medical interventions for each patient were different, which also introduces some bias to our investigations. The mTBI patients requiring neurosurgical operation were not included in our study. More investigations are needed to identify the relationship between nutritional status and clinical outcomes of elderly mTBI patients receiving neurosurgery. Second, the data of patients with injuries on the organ or tissue other than the brain was unavailable. Moreover, most patients who presented to the emergency department did not receive peripheral blood tests, resulting in a loss of data which might influence the outcome of the present research. Third, some important variables, such as alcohol level on admission, substance abuse history, socioeconomic status, psychiatric history, postinjury support, and postinjury GNRI were unavailable, which limits a deeper analysis. Fourth, the pre-morbid functional status also plays an important role in the prognosis of elderly mTBI patients. To reveal the association between nutritional status and recovery of mTBI patients clearly, more future investigations are still needed to evaluate and analyze the pre-morbid functional status. Fifth, 14 adjustment variables were considered in the multivariate logistic regression while 97 patients suffered incomplete recovery, which might invoke the risk of overfitting (55). Moreover, although the study utilized evidence from prior investigations to build the multivariable model, there remain some uncontrolled factors, such as lesion locations, which may confound the outcome. More investigations are still needed to validate the models established in our study. Sixth, the timing of obtaining the peripheral blood sample was different for different patients, which might introduce bias to our outcome. Finally, the elderly populations enrolled in our research were only from a single center; thus, these findings may not apply to other regions. A multicenter prospective investigation with a large sample is still needed to verify the association between nutrition and recovery of geriatric mTBI patients.

## Conclusion

A high GNRI was associated with incomplete recovery in elderly mTBI patients. As a simple and accurate measure of nutritional status, GNRI should be given more attention because it may have an important role in the prognostication of recovery in geriatric mTBI patients. Future investigations are needed to identify nutritional supplementation strategies for older mTBI patients with malnutrition.

## Data availability statement

The raw data supporting the conclusions of this article will be made available by the authors, without undue reservation.

## Ethics statement

This study was approved by the Institutional Ethics Review Board of Beijing Tiantan Hospital (approval number: KY2020-094-02). The Ethics Committee waived the requirement of written informed consent for participation.

## Author contributions

LW and WL: conceptualization and methodology. BZ: data curation, visualization, and writing the original draft. YO: investigation. XG: validation. LW: writing—review and editing. All authors contributed to the article and approved the submitted version.
